# Familiar Strategies Feel Fluent: The Role of Study Strategy Familiarity in the Misinterpreted-Effort Model of Self-Regulated Learning

**DOI:** 10.3390/jintelligence10040083

**Published:** 2022-10-12

**Authors:** Jessica A. Macaluso, Ramya R. Beuford, Scott H. Fraundorf

**Affiliations:** 1 Learning Research and Development Center, University of Pittsburgh, Pittsburgh, PA 15260, USA; 2 Center for the Neural Basis of Cognition, University of Pittsburgh and Carnegie Mellon University, Pittsburgh, PA 15213, USA; 3 Department of Biological Sciences, Harris-Stowe State University, St. Louis, MO 63103, USA

**Keywords:** mediation, metacognition, self-regulated learning, judgments of learning, interleaving effect, education

## Abstract

Why do learners not choose ideal study strategies when learning? Past research suggests that learners frequently misinterpret the effort affiliated with efficient strategies as being indicative of poor learning. Expanding on past findings, we explored the integration of study habits into this model. We conducted two experiments where learners experienced two contrasting strategies—blocked and interleaved schedules—to learn to discriminate between images of bird families. After experiencing each strategy, learners rated each according to its perceived effort, learning, and familiarity. Next, learners were asked to choose which strategy they would use in the future. Mediation analyses revealed, for both experiments, that the more mentally effortful interleaving felt, the less learners felt they learned, and the less likely learners were to use it in future learning. Further, in this study, strategy familiarity predicted strategy choice, also mediated by learners’ perceived learning. Additionally, Study 2 verified that, in contrast to learners’ judgments, the less familiar interleaving schedule resulted in better learning. Consequently, learners are making ineffective learning judgments based on their perceptions of effort and familiarity and, therefore, do not make use of optimal study strategies in self-regulated learning decisions.

## 1. Introduction

As students progress into higher grades, they are tasked with taking charge of more of their own learning, and self-regulation skills become increasingly important (Poropat 2009; Yeager et al. 2014). Ergo, learners must make their own self-regulated learning decisions regarding how to study, such as planning the order in which to study information and making judgments about ideal strategy utilization. As we review in more detail below, laboratory work in cognitive psychology has indicated that some learning strategies, such as spacing out material over time or practicing retrieval, are normatively more effective for learning than others, such as “cramming” in a single block of study or re-reading (Benjamin and Tullis 2010). However, learners are typically not taught the best study methods (Geller et al. 2018; Hartwig and Dunlosky 2012; Kornell and Bjork 2007; McCabe 2011) and, therefore, may choose suboptimal study strategies when studying. While a significant amount of research has been dedicated to determining what methods are best for learning and retention, more information is needed to understand why learners often do not use these optimal learning strategies when studying.

Here, we tested whether study habits and familiarity can explain some of these choices by modeling their role in self-regulated learning decisions. We adapted a paradigm introduced by Kirk-Johnson et al. (2019), and building on prior research by Yan et al. (2016), in which learners experienced two contrasting study strategies—here, interleaved and blocked schedules, which we describe below—and rated each on multiple dimensions before selecting one of the two strategies for future use. We collected ratings of strategy familiarity for the first time in this paradigm, and we considered two ways in which familiarity may relate to the choice of learning strategy: learners prefer habitual strategies simply because they are habitual, or learners interpret habitual strategies as being more effective for learning.

### 1.1. Metacognitive Theory

Metacognitive theory holds that effective learning requires good decisions about how, what, and when to study (Schraw and Moshman 1995). These decisions culminate into a process of self-regulated learning, in which a learner plans for a task, monitors their progress and performance on said task, and then reflects on the outcome. This process is a cycle and continues as a learner uses their personal reflections to modify their behavior and prepare for the next task they will encounter. Self-regulating learning theory holds that the management and selection of efficient study strategies promotes successful learning (Bjork et al. 2013; Dunlosky and Metcalfe 2009; Tullis and Benjamin 2011; Winne and Hadwin 1998; Zimmerman 2000).

The influential framework of Nelson and Narens (1990) divides metacognition into (a) monitoring one’s knowledge and performance and (b) control of activities to achieve one’s desired level of performance. Metacognitive monitoring involves one’s overall awareness and understanding of their own thought processes (Borkowski 1992; Dunlosky and Metcalfe 2009; Flavell 1979; Thiede et al. 2003) and is critical to a learner’s ability to assess their comprehension and task performance (e.g., accuracy). If learners have poor monitoring abilities, they may think that they have mastered a topic, but actually know very little about it (e.g., the Dunning-Kruger effect; Dunning 2011; Kruger and Dunning 1999), or vice versa.

In contrast, metacognitive control represents a learners’ ability to make effective decisions about the necessary steps to complete a task (Corno 1986; Nelson and Narens 1990). Metacognitive control requires focus, an ability to set realistic goals, and being capable of controlling one’s negative or debilitating emotional states, such as performance anxiety. Learners exert metacognitive control, for instance, by spending additional time studying difficult material (Dunlosky and Hertzog 1998; for review, see Son and Metcalfe 2000), or by using distributed rather than massed practice to study material (Baddeley and Longman 1978; Benjamin and Bird 2006; Pyc and Dunlosky 2010; Son 2004, 2010; Toppino et al. 2009). Critically, successful metacognitive monitoring is necessary for effective metacognitive control—there is evidence that learners’ study decisions (i.e., their control) are causally related to their metacognitive monitoring (Metcalfe and Finn 2008). Therefore, if learners do not accurately monitor their learning, they are unlikely to make successful self-regulated learning decisions.

### 1.2. Metacognitive Illusions and the Interleaving Effect

Is self-regulated learning typically successful? Although metacognitive monitoring can be reasonably accurate in many cases (e.g., Benjamin et al. 1998; Wixted and Wells 2017), it is also subject to some systematic biases and errors. For one, learners are not typically taught which study methods actually engender the most learning (Geller et al. 2018; Hartwig and Dunlosky 2012; Kornell and Bjork 2007; McCabe 2011). In addition, researchers have found that learners do not have privileged, or direct, access to the strength of their own learning and memory beyond what they can retrieve (Koriat 1995, 1997). Learners instead must make inferences about how much they feel they are learning by assessing their environment and their internal state (Koriat 1997; Schwartz et al. 1997). These learners’ own judgements regarding memory of information are also referred to as judgments of learning (JOLs; Nelson and Dunlosky 1991).

As JOLs are inferential, prior work has found that they can be subject to metacognitive illusions, in which learners give lower judgments regarding their learning of material when using strategies that, in reality, are more efficient for learning, such as retrieval practice (Karpicke and Roediger 2008; Karpicke 2009; Tullis et al. 2013), repeated study (Koriat et al. 2002; Kornell and Bjork 2008a; Kornell et al. 2011), distributed practice (Logan et al. 2012), and generation from a prompt (Besken 2016).

In the present study, we focus on one such illusion that has received particular attention in the literature: learners’ preference for a blocked schedule over an interleaved schedule when ordering to-be-learned material. An interleaving schedule involves learning study items from various categories in an intermixed fashion, such as learning words from multiple topics, such as animals, foods, and colors. In contrast, a blocked schedule involves a learner studying items within a single topic (e.g., *t*-tests or finches) before moving onto the next topic (e.g., ANOVAs or magpies).

In multiple studies of objective learning, interleaving has proven to be an advantageous learning strategy for a multitude of subjects, including words and visual materials such as bird species or paintings (Brunmair and Richter 2019; Kang and Pashler 2012; Kornell and Bjork 2008a; Kornell et al. 2010; Metcalfe and Xu 2016; Verkoeijen and Bouwmeester 2014; Wahlheim et al. 2011, 2012; Yan et al. 2016; Zulkiply et al. 2012; Zulkiply 2015; Zulkiply and Burt 2013a, 2013b). In their meta-analysis of the interleaving effect, Brunmair and Richter (2019) found robust evidence that interleaving is more effective than blocking, with an intermediate effect size (Hedges’ *g =* 0.42).

It is important to note that interleaving is not necessarily always best for learning. In their meta-analysis, Brunmair and Richter (2019) identified several moderators of the interleaving effect, such as the type of learning material, material characteristics, retention interval length, retention of previously learned materials versus a transfer test, successive versus simultaneous presentation, and temporal spacing. Prior research obtained mixed results with respect to learning artificial objects and mathematical concepts (Carvalho and Goldstone 2014a; Dobson 2011; Higgins and Ross 2011; Lavis and Mitchell 2006; Mitchell et al. 2008; Noh et al. 2016; Rau et al. 2010; Zulkiply and Burt 2013a, 2013b). Interleaving was found to be most effective for learning material that shows subtle, rather than pronounced, differences between categories (Carvalho and Goldstone 2014a, 2015, 2017; Zulkiply and Burt 2013b). One study found an interaction between retention interval length, where interleaving was more beneficial for a thirty-day retention interval compared to a one-day retention interval (Rohrer et al. 2015), but more research is necessary to explore how retention interval length impacts the benefits of interleaving. However, prior research has found that interleaving is beneficial when differentiating between naturalistic images, such bird families (Birnbaum et al. 2013; Kirk-Johnson et al. 2019; Wahlheim et al. 2011), as in our present study.

Interleaved practice is thought to facilitate learning in these circumstances because it promotes discrimination between subsequent items (*discriminative contrast hypothesis*; Kang and Pashler 2012). The attentional bias framework (Carvalho and Goldstone 2014b) expands on the discriminative contrast hypothesis by suggesting that interleaving calls attention to differences between exemplars, while blocking brings focus to similarities. Consequently, interleaved practices are assumed to be superior for inductive learning—where a learner acquires rules by viewing examples (e.g., in the present studies, discriminating between bird families) (Michalski 1983)—especially when these categories are low in discriminability and are very similar to each other (for review, see Brunmair and Richter 2019; Firth et al. 2021). Interleaving is particularly helpful in inductive learning.

Despite the normative benefits of interleaving, prior studies found that learners often prefer to employ a blocked schedule and feel that they learn more from blocking, even when the same learners were shown to objectively learn more from interleaving (e.g., Kirk-Johnson et al. 2019; Kornell and Bjork 2008a; Kornell et al. 2010; Tauber et al. 2013; Wahlheim et al. 2012; Yan et al. 2016; Zulkiply et al. 2012). That is, learners experience errors in their metacognitive monitoring due to inaccurate judgments regarding the effectivity of interleaving. This was viewed as a *metacognitive illusion* (Serra and Metcalfe 2009), which likely causes learners to not self-regulate their learning, or not do so in the most advantageous way (Zimmerman 2008).

These metacognitive illusions are of interest for multiple reasons. First, the discrepancy between learners’ monitoring and normative efficacy reveals the mechanisms of metacognitive monitoring and how and why this may diverge from objective reality. Second, as a practical matter, because monitoring learning has a causal influence on self-regulated learning decisions, these errors in metacognitive monitoring are likely to have consequences for learning, in that learners do not use the best study strategies in self-regulated learning decisions.

### 1.3. The Misperceived-Effort Hypothesis

Why do people not always recognize the value of, and do not employ, interleaving and other effective learning strategies? Kirk-Johnson et al. (2019) proposed a *misinterpreted-effort hypothesis* of these metacognitive illusions, which draws on two established strands of research in human learning and memory.

First, there is ample evidence that learners often make JOLs using an ease-of-processing heuristic, whereby individuals judge information that is easy to process in the moment as information that is well-learned, whereas information that requires more mental effort is judged as not being well-learned (Anand and Sternthal 1990; Begg et al. 1989; Kornell et al. 2011; Rhodes and Castel 2008; Undorf and Erdfelder 2011). Although reasonable in many cases, the heuristic nature of this inference means that it is not always right (Benjamin et al. 1998); in some cases, mental effort shows growth rather than a poor ability to learn (Susser et al. 2016). Second, the principle of desirable difficulties (Bjork 1994; Bjork and Bjork 1992; Schmidt and Bjork 1992) holds that, contrary to the ease-of-processing heuristic, many strategies effective for long-term learning require more initial effort.

Given these findings, Kirk-Johnson et al. (2019) suggested that one challenge in self-regulated learning is that strategies effective for long-term learning and retention often require more initial effort, but learners often misinterpret this mental effort as being a sign of poor learning. Consequently, they do not choose to employ these strategies.

Past work has found evidence for this misinterpreted-effort model. In Study 1 of Kirk-Johnson et al. (2019), participants learned to discriminate among six bird families, with images presented one at a time. Participants learned via two different and contrasting study schedules: blocked and interleaved. A blocked schedule requires one to study exemplars that are grouped together by category, such as a particular bird family (e.g., all the finches, then all the jays, and then all the orioles) while an interleaved schedule requires one to study exemplars from multiple categories mixed together (e.g., intermixed sparrows, tyrant flycatchers, and wood warblers).

**Figure 1:**
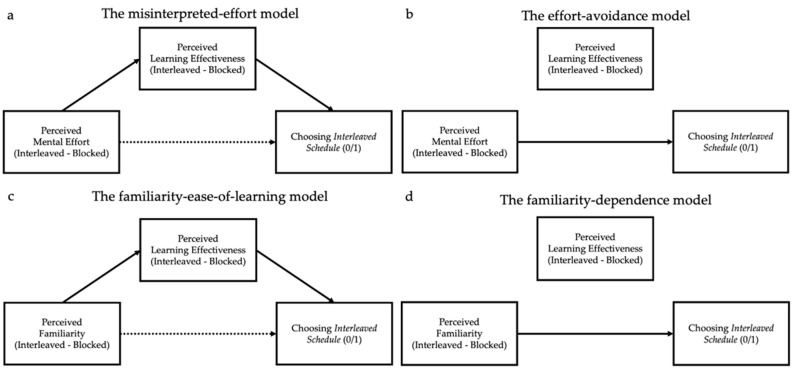
(**a**) Theoretical model of a mediation analysis, focusing on the role of perceived mental effort on perceived learning effectiveness on strategy choice (**b**) or a direct link between perceived mental effort to strategy choice, independent of perceived learning; (**c**) theoretical model of a mediation analysis focusing on the role of perceived familiarity on perceived learning effectiveness on strategy choice (**d**) or a direct link between perceived familiarity to strategy choice, independent of perceived learning.

After experiencing each of these two strategies, participants filled out self-report questionnaires to assess (a) their perceptions of the mental effort required by the strategy (perceived effort) and (b) their perceptions of how well they had learned the material (perceived learning). While learners believed they were learning more from the blocked schedule, prior and present work has found that learners actually learn better with an interleaved schedule (Kornell and Bjork 2008a; Kornell et al. 2010; Tauber et al. 2013; Wahlheim et al. 2012; Yan et al. 2016; Zulkiply et al. 2012), establishing metacognitive failure. What accounts for this error? Critically, mediation analyses supported the misperceived-effort hypothesis: the more mentally effortful the participants found the interleaved schedule compared to the blocked schedule, the less effective they felt the interleaved schedule was for their learning, and the less likely they were to choose an interleaved schedule for their future studies (see Figure 1a for a theoretical model). Kirk-Johnson et al. (2019) replicated this pattern in several subsequent studies that extended the misinterpreted-effort hypothesis to other choices in self-regulated learning (the contrast between retrieval practice and re-reading).

These results stand in contrast to another possibility: perhaps learners avoid effortful strategies simply because they are effortful, and they want to “take the easy way out”. This hypothesis predicts a direct effect of perceived effort on strategy choice, independent of perceived learning. Kirk-Johnson et al. (2019) found some evidence for such an effect, their effort-avoidance hypothesis, but only in participants’ retrospective ratings rather than their immediate perceptions, and only in two of three studies, whereas the indirect effect predicted by the misinterpreted-effort hypothesis was present throughout. Overall, Kirk-Johnson et al.’s (2019) results suggest that people do not merely avoid strategies because they are effortful; they try to choose the best strategy, but the visceral experience of mental effort confuses them as to which strategies—e.g., blocking vs. interleaving—are best for learning.

### 1.4. Habits and Familiarity in Self-Regulated Learning

Above, we discussed how interpretations of mental effort could account, at least in part, for the metacognitive illusions whereby learners do not appreciate the value of, and do not choose, strategies that are normatively more effective for learning. However, other variables are likely to influence self-regulated learning decisions and could—at least in principle—also contribute to these metacognitive illusions. Here, we consider the role of familiarity and study habits.

In general, familiarity is likely to affect how well learners believe they are learning information; people tend to favor information, habits, or things that are familiar to them (the familiarity heuristic; Metcalfe et al. 1993). Habitual study strategies are likely to feel more familiar, and thus learners may prefer these habitual strategies because they are well-acquainted with them (Ariel and Dunlosky 2013).

Indeed, recent laboratory work provides evidence for the role of habits in self-regulated learning decisions. Ariel et al. (2011; see also Dunlosky and Ariel 2011) examined the role of one particular habit: the tendency to read in a particular direction. They found that English speakers tended to study material (in this case, foreign-language vocabulary words) in a left-to-right order, regardless of difficulty, consistent with the left-to-right reading direction in English. By contrast, speakers of Arabic, a language that reads right-to-left, tend to study material going right-to-left, regardless of difficulty. (We note, however, that while it can be reasonably assumed that speakers of English versus Arabic have different reading habits, Ariel et al. (2011) did not directly survey participants regarding their study or reading habits or assess the familiarity of these two approaches.)

Could a preference for habitual or familiar study strategies contribute to some of the metacognitive illusions discussed above? We argue that they might. Scientists of human learning and memory have noted that the structure and practice of formal education do not always align with what is necessarily optimal for learning and retention (e.g., Roediger and Pyc 2012). For example, despite the benefits of interleaving, topics in courses are often blocked (e.g., two weeks of *t*-tests in a statistical course, followed by two weeks of correlations) rather than interleaved (perhaps for understandable practical reasons). Thus, it is quite plausible that learners have acquired suboptimal study habits and that strategies that are normatively effective for learning are not necessarily familiar to them, including in the case of interleaving.

Indeed, habits and familiarity could contribute to self-regulated learning decisions in at least two ways. First, learners could rely on existing study habits, even when they are not optimal for learning, simply because they are habitual. Habits are less cognitively demanding and less time-consuming than more effortful, agenda-based processes (Ariel et al. 2009; Winne and Hadwin 1998). Research assessing the effects of time constraints on decision making and reasoning indicates that people typically rely on quick, heuristic-based reasoning instead of more analytical and logical processes when decision-making is restricted (Evans and Curtis-Holmes 2005; Roberts and Newton 2001; Schroyens et al. 2003). For example, prior work has also found that when learners are given an opportunity to self-regulate their own learning during a category-learning task of classifying birds, participants spend most of their study time blocking the need-to-be-learned exemplars (Tauber et al. 2013). Second, similar to the misperceived-effort hypothesis discussed above, learners may perceive the unfamiliarity of a strategy as a sign that it is ineffective for learning. For instance, the fact that most courses present material in a blocked rather than an interleaved fashion may be taken as an implicit endorsement of the value of blocking for learning (whether justified or not), and the unfamiliarity of an interleaving schedule may be interpreted by learners as a sign that it is not ideal for learning. Returning to the English speakers in the study of Ariel et al. (2011), discussed above, we do not know if the participants chose the left-to-right way of studying material simply due to habitual reading bias or if people genuinely believed that they would learn more by reading left-to-right.

### 1.5. Present Work

In the present study, we tested the role of habits in learners’ study decisions, focusing on the decision of how to-be-learned material should be ordered to maximize learning. We adapted the paradigm introduced by Kirk-Johnson et al. (2019), with the addition of measures of perceived familiarity. Learners experienced two different learning strategies and rated each one for its perceived mental effort, perceived learning, and perceived familiarity before selecting one of those strategies for future learning; this paradigm allowed for us to assess how each of these perceptions related to each other and to strategy choice. Perceptions of effort, learning, and familiarity were explicitly evaluated via self-report questionnaires. Consistent with Kirk-Johnson et al. (2019), these perceptions were collected immediately following each individual study strategy, which is typical when measuring visceral experience (Loewenstein 1996), as well as retrospectively for a semantic-differential comparison of both strategies (as was carried out with other metacognitive studies; Kornell and Bjork 2008b; Kornell et al. 2010; Yan et al. 2016; Zulkiply et al. 2012). After experiencing and providing perception ratings for both strategies, participants chose which of the two strategies they would prefer to use in the future for novel material.

We tested two competing hypotheses about how habits could influence self-regulated learning behavior. Under the *familiarity-dependence hypothesis,* learners prefer familiar strategies, regardless of what they feel they are learning from, simply because familiarity leads participants to do what is habitual. This hypothesis predicts that, within a mediation analysis, we should see a direct effect of familiarity on strategy choice—that is, one not mediated by perceived learning. By contrast, under the *familiarity-ease-of-learning hypothesis,* strategy familiarity contributes to the feeling of learning due to the fact that a study strategy is familiar and habitual to them. This hypothesis predicts an indirect effect of familiarity on choice, mediated by perceived learning. (See Figure 1b for the competing predictions of these two models).

This approach makes several contributions to our understanding of self-regulated learning and the role of habits therein. First, we expand our understanding of the metacognitive illusions discussed above by testing whether familiarity may contribute to them. Second, we depart from several previous laboratory studies by including an explicit measure of participants’ habits that does not require us to assume which strategy is likely to be more familiar to participants. Third, we go beyond establishing the existence of a habit effect by contrasting two competing mechanistic hypotheses about why habits affect self-regulated learning decisions. Finally, although not our primary aims, collecting measures of perceived mental effort, perceived learning, and strategy choice also allows for us to attempt to replicate Kirk-Johnson et al. (2019) regarding the impact of mental effort on perceived learning with final study strategy choice.

The two experiments that we report are extremely similar, with the only difference being that Study 2 included an additional post-test to verify that an interleaving schedule yielded better learning for the present materials. Consequently, we present the materials and methods sections of both experiments together, followed by the results of Study 1 and Study 2. The paper will end with a joint discussion section regarding the results of both studies.

## 2. Materials and Methods

### 2.1. Study 1

#### 2.1.1. Participants

To determine an adequate sample size for both of our studies, we used the mediation analysis of perceived mental effort by Kirk-Johnson et al. (2019) to estimate a potential effect size for the effects of familiarity. A power analysis using R package *powerMediation* (Qiu 2021) indicated *N* = 96 would achieve 95% power to detect a significant mediation effect at α = .05 for both perceived mental effort and perceived familiarity.

Therefore, we originally targeted *N* = 96; however, we continued to run participants until the end of the term to fulfill a departmental need for research participation opportunities. Post-hoc power analyses found that the observed sample size achieved a power of greater than 99%.

A total of 328 participants were recruited via Sona Systems and received partial course credit for their one-time survey completion. Participants were required to speak fluent English, be 18 years of age or older, and have access to a computer (71.4% female; 71.7% White, 9.1% Black/African American, 23.7% Asian, and 1.8% American Indian or Alaskan Native; 8.8% Hispanic or Latino; 98.5% 18–24, 0.6% 25–34, 0.3% 35–44, and 0.3% 45–54; 1.5% some high school, 41.6% high school diploma, 55% some college, 1.2% bachelor’s degree, and 0.3% some graduate school).

#### 2.1.2. Materials

##### Learning Materials

The experiment began with two practice phases—one with a blocked schedule and the other with an interleaved schedule—that acquainted participants with the two schedules that would be used in the future study phases prior to presenting the critical to-be-learned material. In each practice phase, participants received two bird families with three images of each (six images per phase, twelve images total): penguins and flamingos in the first practice block, and parrots and ducks in the second. These four bird families were used in the practice phase because they were expected to be familiar and easy to identify, rather than the birds in the more difficult study phase. These images were obtained by Kirk-Johnson et al. (2019) from a stock photo website and varied in size but were comparable to the study phase stimuli.

Next, participants underwent a demonstration phase, where they experienced one picture of each bird family (six in total) that they would encounter during the study phase. In the two study phases, participants learned to distinguish between three different bird families per each study phase. These images were also obtained from Kirk-Johnson et al. (2019) and were originally selected by Tullis and Benjamin (2011). Each image consisted of a color picture of a bird with dimensions of 322 × 403 pixels. In one study phase, participants learned to distinguish between finches, jays, and orioles. In the other, participants learned to distinguish between sparrows, tyrant flycatchers, and wood warblers. Each study phase was composed of fifteen photographs of each bird family (forty-five birds total). Following the study phase, there was a final wrap-up phase, where a single image of each bird family was used.

##### Immediate-Perception Questionnaire

We used questionnaires to evaluate participants’ experiences after completing both the blocked and interleaved schedules. Questionnaire items were assessed on a Likert scale rating from one to six, where one indicated low endorsement and six indicated high endorsement (see Appendix A.1 for survey items).

Measures of perceived mental effort and perceived learning were taken directly from Kirk-Johnson et al. (2019) to facilitate comparisons between studies and because those measures were found to have good reliability. Four questionnaire items evaluated the participants’ perceived mental effort regarding each strategy (e.g., How mentally exhausting was the last exercise? 1 = Not at all, 6 = A lot); four evaluated participants’ perceived learning of a strategy (e.g., How good do you think your memory for the different types of birds will be? 1 = Not very good, 6 = Extremely good). In this study, for the first time, we constructed four novel questionnaire items in a similar format to evaluate participants’ perceived familiarity of a strategy (e.g., How much did the last exercise resemble the structure of your classes? 1 = Not well, 6 = Extremely well). Participants completed each of the three questionnaires twice: once for the blocked schedule and once for the interleaved schedule.

We averaged the immediate-perception questionnaire items to the six produced composite scores: perceived mental effort of the blocked schedule (α = .76), perceived mental effort of the interleaved schedule (α = .78), perceived learning from the blocked schedule (α = .91), perceived learning from the interleaved schedule (α = .93), perceived familiarity with the blocked schedule (α = .83), and perceived familiarity with the interleaved schedule (α = .76). Due to a Cronbach alpha of above .70, we found good reliability for our novel measure of perceived familiarity. For the composite scores, lower scores indicated low endorsement and higher scores indicated high endorsement.

##### Retrospective Semantic-Differential Questionnaire

For both additional converging evidence and consistency with previous research that used end-of-experiment judgments (Kirk-Johnson et al. 2019; Kornell and Bjork 2008b; Kornell et al. 2010; Yan et al. 2016; Zulkiply et al. 2012), we also used a second questionnaire to assess participants’ reflective comparisons of the two strategies. This retrospective semantic-differential questionnaire comprised of twelve semantic-differential items evaluating participants’ comparative perceptions of the two study schedules on a continuum, where one indicated the blocked schedule and six indicated the interleaved schedule (e.g., Which do you think is a more effective learning strategy for you? 1 = Grouped Together, 6 = Not Grouped Together).

As with the immediate-perception measures, we took the items measuring perceived mental effort and perceived learning directly from Kirk-Johnson et al. (2019) on the basis of continuity and their established reliability. Four questionnaire items evaluated perceived mental effort (α = .83) and four evaluated perceived learning (α = .93). We then created four new items evaluating perceived familiarity (α = .84) (see Appendix A.2 for survey items). The novel measure of perceived familiarity expands on previous work by integrating the impact of familiarity into the misinterpreted-effort model (Kirk-Johnson et al. 2019).

#### 2.1.3. Procedure

**Figure 2:**
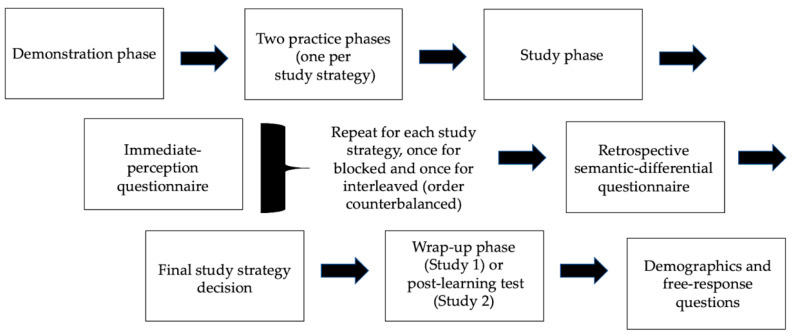
Flow of study activities in Study 1 and Study 2.

The experiment took place on the Qualtrics online survey platform from the participants’ location of choice. The overall experiment was composed of a demonstration phase, two practice phases, two study phases, which were each followed by the immediate-perception questionnaire, the retrospective semantic-differential questionnaire that came after participants experienced both strategies, a final decision between the two study strategies, a wrap-up phase (or post-test for Study 2), a collection of demographic information, and a couple of questions regarding possible technical difficulties and any thoughts that participants had on the experiment (see Figure 2).

First, prior to learning, participants experienced an untimed demonstration phase in which they were shown a bird photograph from each of the six different bird families used in the study sessions, one at a time and chose the bird family to which the bird belonged (i.e., Which type of bird is this?). All participants viewed the demonstration phase bird images in the same order. The demonstration phase was included to help establish the information that participants needed to learn during the study phases of the experiment. Participants selected a bird family from one of three presented options (i.e., either finch, jay, or oriole or sparrow, tyrant flycatcher, or wood warbler?). After participants chose their answer, the correct response was shown (e.g., “The correct answer is Sparrow”.), and the experiment continued to the next bird image. This demonstration phase helped to establish to participants that identifying bird families is not intuitive and that studying and learning is necessary to be able to distinguish between bird families. Mean performance indicated that 65% of the practice items were answered correctly, and only 6% of participants answered all six practice questions correctly^1^. This established that most people did not know these bird families prior to the experiment.

Following the demonstration phase, participants went through two practice phases (one for blocked and one for interleaved) in which they learned the two study strategies prior to the main study phase. A counterbalanced design was used where half of the participants were randomly assigned to receive the blocked schedule first, and half were assigned to receive the interleaved schedule first. Rather than using jargon, participants were told that they would study bird families “grouped together by type” (blocked study) and “not grouped together by type” (interleaved study). With each practice phase, all participants were shown two bird families with three images of each (penguins versus flamingos in the primary block, and parrots versus ducks in the secondary block). For the primary block, participants who were assigned to a blocked schedule first saw penguins and flamingos blocked while participants who were assigned to an interleaved schedule first saw penguins and flamingos interleaved. For the secondary block, participants who first saw a blocked schedule next saw parrots and ducks interleaved and participants who first saw interleaved saw parrots and ducks blocked. Under both conditions, each photograph was displayed one at a time, for three seconds each, and the name of the bird family was included above each bird picture. The task automatically moved to the next bird image after three seconds had passed. Exemplars were not randomized by participant; that is, regardless of whether the categories were blocked or interleaved, among the flamingo exemplars seen by participants, the first flamingo was always the same image, as was the second, and so on.

After both practice phases were completed, participants saw a text screen informing them that they were starting the actual study, that they will study birds that they will later be tested on, and that they will not be able to go back at any point during the experiment.

Participants then began the main study phases. For the first portion of the study phase, participants studied three different bird families with fifteen examples of each bird family (forty-five images in total). For the second portion of the study phase, participants did the same as with the first part, but studied using the alternative study strategy and three different bird families. The order of study strategies was counterbalanced across participants, such that approximately half of the participants (*n* = 179) experienced the blocked schedule first, then the interleaved schedule, and the other participants (*n* = 148) experienced the interleaved schedule first, then the blocked schedule. All the participants studied finches, jays, and orioles in the first study phase, regardless of study strategy order assignment (i.e., using blocking first or interleaving first), and all the participants studied sparrows, tyrant flycatchers, and wood warblers in the second study phase regardless of study strategy order assignment (i.e., using interleaving second or blocking second). In the blocked condition, participants saw all the exemplars of one bird family (finches or sparrows), then all the examples of the next, and so on; the order of families was constant. In the interleaved condition, participants rotated through seeing one exemplar of each family. As with the practice phases, the task automatically moved to the next bird image after three seconds had passed, and exemplars were not randomized by participant.

Immediately following the completion of each study phase, participants completed the untimed immediate-perception measures, first for perceived mental effort, then perceived learning, and lastly for perceived familiarity for the utilized strategy. For example, participants experienced the blocked schedule and next completed the immediate-perception assessment measures for the just-completed blocked strategy, then participants experienced the interleaved schedule and then completed the immediate-perception assessment measures for the just-completed interleaved strategy. Within each category (e.g., mental effort), the four Likert scaled measures were not randomized; they were in the same order for all participants, regardless of condition.

After experiencing and rating each strategy using the immediate-perception assessment measures, participants completed the untimed retrospective semantic-differential questionnaire to evaluate the perceptions of the two different strategies in relation to each other, first for perceived mental effort, then for perceived learning, and lastly for perceived familiarity. As with the immediate-perception measures, within each category (e.g., mental effort), the four Likert scaled measures were not randomized; they were in the same order for all participants, regardless of condition.

Following the completion of the retrospective semantic-differential questionnaire, participants chose which strategy they would (hypothetically) prefer for learning a novel set of bird families. Participants were queried, “Imagine that you had to study more birds like you did today. Which strategy would you use to study the types of birds, so you would be able to take a test on them later?” Participants then answered a two-choice question item by selecting either “Grouped together” for a blocked schedule or “Not grouped together” for an interleaved schedule.

Following the question regarding study strategy choice for future use, participants were shown a screen of text explaining that most people do better with interleaved practice: “Several prior studies in cognitive psychology (such as Yan et al. 2016) have found that 90% of people learn better when examples are not grouped together. But learners often fail to appreciate the benefits of this method, because grouping seems easier and leads to greater fluency during study.” and were asked if they would change their answer regarding which strategy they would prefer to use in the future. This was an untimed, open-ended, free-response question that was included on an exploratory basis to see if participants would change which study strategy that they prefer based on the presented evidence that an interleaved strategy is typically superior in comparison with a blocked strategy.

Next, participants went through the untimed wrap-up phase of the experiment. Participants were shown one image of each bird family and asked, “Which type of bird is this?” while being presented with answer options corresponding to the correct bird family as well as the two alternative bird families that were shown within the same study block. The wrap-up phase served to fulfill the expected test, which was mentioned to participants in the instructions of the study. Our interest was in participants’ perception choices rather than their performance on the wrap-up phase test.

Lastly, participants filled out demographic information, such as their gender and race, and answered a question about technical difficulties (Did you experience any technical problems while completing this task? Yes (Please explain) or No). Participants also had the opportunity to share about their experience during the study before officially completing the experiment and approximately two-thirds of participants (67.4%) responded.

### 2.2. Study 2

#### 2.2.1. Participants

A total of 377 participants were recruited via Sona Systems and obtained partial course credit for their one-time study completion. All subjects reported that they spoke fluent English, had access to a computer, and were eighteen years or older (76% female; 68% White, 4% Black/African American, 20% Asian, less than 1% American Indian or Alaskan Native; 6% Hispanic or Latino; 99.7% age 18–24, 0.3% age 25–34; 0.3% some high school, 44% high school diploma, 55% some college, and 1% bachelor’s degree).

#### 2.2.2. Materials

##### Learning Materials

The learning materials were identical to Study 1, with the exception of the wrap-up phase. Instead, Study 2 had a longer test that included the original wrap-up image from Study 1, as well as 10 new images from BirdWeb (http://www.birdweb.org; accessed 15 August 2021). For these new test items, one photo was selected for each of the ten different species within the same family (i.e., one photo of each of ten different finch species).

##### Immediate-Perception Questionnaire

The immediate-perception questionnaire for Study 2 was identical to Study 1. In the same way, we averaged the immediate-perception questionnaire items to produce six composite scores: perceived mental effort of the blocked schedule (α = .81), perceived mental effort of the interleaved schedule (α = .78), perceived learning from the blocked schedule (α = .92), perceived learning from the interleaved schedule (α = .94), perceived familiarity with the blocked schedule (α = .80), and perceived familiarity with the interleaved schedule (α = .70). As with Study 1, we found good reliability for our measure of perceived familiarity (a Cronbach alpha at or above .70).

##### Retrospective Semantic-Differential Questionnaire

The retrospective semantic-differential questionnaire for Study 2 was identical to Study 1: four questionnaire items evaluated perceived mental effort (α = .84), four evaluated perceived learning (α = .94), and four evaluated perceived familiarity (α = .81).

#### 2.2.3. Procedure

The procedure of Study 2 was identical to Study 1, with the exception of the wrap-up phase. Instead, Study 2 had a post-learning test where participants classified eleven novel images of each bird family to assess their learning.

Participants’ mean performance in the demonstration indicated that 63% of the practice items were answered correctly, and only 6% of participants answered all six practice questions correctly^2^.

As with Study 1, during the study phases, the order of study strategies was counterbalanced across participants, where about half of the participants (*n* = 187) experienced the blocked schedule first, followed by the interleaved schedule, and about half of the participants (*n* = 189) experienced the interleaved schedule first, followed by the blocked schedule.

Instead of a wrap-up phase in Study 1, in Study 2, participants classified eleven novel exemplars of each bird family to measure post-test learning; this post-test was untimed. This allowed us to assess which study schedule, blocked or interleaved, participants actually learned best from, regardless of their perceptions of their performance. A total of 68.4% of participants responded to questions about demographic information, technical difficulties, and their overall experience with the task.

## 3. Results

### 3.1. Study 1

#### 3.1.1. Strategy Choice

When given the opportunity to choose to either use the blocked strategy or the interleaved strategy for future use, most participants chose a blocked schedule (76.6%) compared to the interleaved schedule.

#### 3.1.2. Immediate-Perception Questionnaire

**Figure 3:**
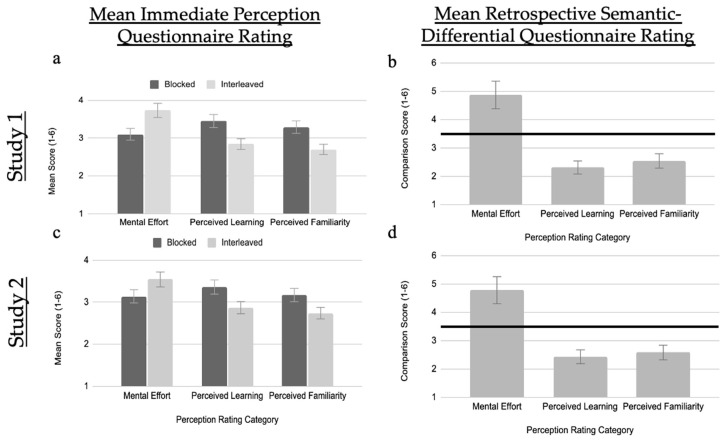
Mean immediate-perception questionnaire ratings for perceived mental effort, perceived learning, and perceived familiarity for the two study strategies for Study 1 (**a**) and Study 2 (**c**); Mean retrospective semantic-differential questionnaire ratings for retrospective perceived mental effort, retrospective perceived learning, and retrospective perceived familiarity when comparing study strategies for Study 1 (**b**) and Study 2 (**d**). 1 indicates a blocked study schedule, 6 indicates an interleaved study schedule. For the retrospective semantic-differential items, the solid black line at 3.5 indicates no preference for one strategy over another.

Why did participants prefer a blocked schedule over an interleaved schedule? One reason for this may be that they perceived the strategies as differing in their required mental effort, their resulting learning, and/or their familiarity. Figure 3a,c show the mean perceptions of the strategies in both experiments.

Participants perceived the interleaved schedule (*M* = 3.73, *SD* = 1.22) as, on average, being 0.63 points more mentally effortful (95% CI: [0.76, 0.51]) than the blocked schedule (*M* = 3.10, *SD* = 1.05), *t*(327) = 10.04, *p* < .001, *d* = 0.55. They also judged the interleaved schedule (*M* = 2.84, *SD* = 1.11) as, on average, being 0.61 points less effective (95% CI: [−0.47, −0.45]) for their learning when compared to the blocked schedule (*M* = 3.45, *SD* = 1.05), *t*(327) = −8.53, *p* < .001, *d* = −0.47. Lastly, they judged the interleaved schedule (*M* = 2.70, *SD* = 0.95) as, on average, being 0.59 points less familiar (95% CI: [−0.45, −0.74]) than the blocked schedule (*M* = 3.29, *SD* = 1.21), *t*(327) = −8.22, *p* < .001, *d* = −0.45.

#### 3.1.3. Retrospective Semantic-Differential Questionnaire

We also explored if the same relations existed in participants’ retrospective judgements about blocked versus interleaved practice (see Figure 3b,d). Recall that these judgements were on a single semantic-differential Likert scale, where blocked practice was one on end (1) and interleaved practice on the other end (6). Thus, we compared participants’ responses to the midpoint of the established scale (3.5). Responses at the midpoint would indicate that participants did not view either strategy as being more effortful, felt that they learned more from either strategy, or did not view either strategy as more familiar. In contrast, a number larger than 3.5 indicates that participants felt that the measure applied more to interleaved, whereas a smaller number indicates that the measure applied more to blocked.

These comparisons showed a similar pattern to the immediate-perceptions: participants judged an interleaved schedule as being significantly more effortful than a blocked schedule (*M* = 4.87, *SD* = 1.23, *t*(327) = 20.06, *p* < .001, 95% CI: [4.73, 5.00], *d* = 1.11), as significantly less effective for learning (*M* = 2.31, *SD* = 1.45, *t*(327) = −14.85, *p* < .001, 95% CI: [2.15, 2.47], *d* = −0.82), and as significantly less familiar (*M* = 2.54, *SD* = 1.16, *t*(327) = −14.97, *p* < .001, 95% CI: [2.42, 2.67], *d* = −0.83).

#### 3.1.4. Relation of Immediate Perceptions to Strategy Choice

Our critical dependent measure was participants’ choice between a blocked schedule (coded as 0) and an interleaved schedule (coded as 1), and our predictors of interest were the differences between participants’ perceptions of blocked versus interleaved schedules. Consequently, we constructed three difference scores: one for perceived mental effort, one for perceived learning, and one for perceived familiarity. Positive values on the created scales indicates that participants perceive the interleaved schedule as being more effortful, more effective for learning, or more familiar.

Individual logistic regression analyses showed that for every point that participants perceived the interleaved schedule as being more mentally effortful (relative to a blocked schedule), their odds of choosing it for future study declined by 0.53 times (95% CI: [0.41, 0.70]), Wald *z* = −4.61, *p* < .001, Nagelkerke *R*^2^ = .12. By contrast, for every point that participants perceived the interleaved schedule as being more effective for learning, their odds of choosing it increased by 4.25 times (95% CI: [2.96, 6.07]), Wald *z* = 7.89, *p* < .001, Nagelkerke *R*^2^ = .43, and for every point they perceived interleaving as being more familiar, their odds of choosing it increased by 1.42 times (95% CI: [1.14, 1.76]), Wald *z* = 3.13, *p* = .002, Nagelkerke *R*^2^ = .06. Given these bivariate relations, we next tested our key hypotheses that the effects of perceived effort and perceived familiarity on strategy choice were mediated by perceived learning.

For all mediation analyses in this paper, we conducted a bootstrapped indirect effect analysis with 1000 samples using the R package *mediation* (Tingley et al. 2014) and report unstandardized direct, indirect, and total effects. We tested two mediation models: one testing whether perceived learning mediated the effect of perceived mental effort on final study strategy choice, and the other testing whether perceived learning mediated the effect of perceived familiarity on study strategy choice, resulting in a total of four models. These models test our hypotheses: whether perceived mental effort impacts perceived learning, and thus impacts final study strategy choice (interleaved or blocked), and whether perceived familiarity impacts perceived learning, and thus impacts final study strategy choice (interleaved or blocked).

Mediation was inferred if the 95% confidence interval for the critical indirect parameter (i.e., perceptions of effort or familiarity on strategy choice through the mediator of perceived learning) did not include zero.

**Figure 4:**
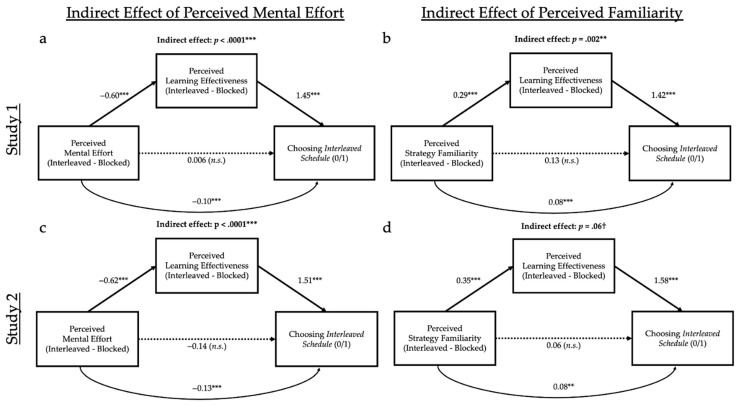
Bootstrapped mediation analyses for Study 1 for the unstandardized effects of mental effort (**a**) and familiarity (**b**) using the immediate-perception measures; bootstrapped mediation analyses for Study 2 for the unstandardized effects of mental effort (**c**) and familiarity (**d**) using the immediate-perception measures. † < .10, ** *p* < .01, *** *p* < .001.

Mediation analyses (see Figure 4a), replicating Kirk-Johnson et al. (2019) and supporting our familiarity-ease-of-learning hypothesis, revealed the more learners perceived interleaving as effortful, the less they perceived they learned (*t* = −11.31, *p* < .001, *R*^2^ = .28, and consequently they were less likely to choose this method (*z* = 7.35, *p* < .001, partial *R*^2^ = .36; indirect effect: *z* = −4.61, *p* < .001). The direct effect of perceived mental effort on strategy choice was insignificant (*z* = 0.03, *p* = .97, partial *R*^2^ < .01), suggesting full mediation.

Critically, as hypothesized, strategy familiarity had a similar indirect effect on choice (see Figure 4b), which was also mediated by perceived learning (*ps* < .01): the more learners perceived interleaving as being less familiar, the less they perceived they learned (*t* = 5.54, *p* < .001, *R*^2^ = .09), and consequently the less likely they were to choose this method (*z* = 7.66, *p* < .001, partial *R*^2^ = .40; indirect effect: *z* = 3.13, *p* = .002). The direct effect of perceived familiarity on strategy choice was also insignificant (*z* = 0.86, *p* = .39, partial *R*^2^ < .01), again suggesting full mediation.

#### 3.1.5. Relation between Retrospective Semantic-Differential Perceptions and Choice of Final Strategy

We also examined whether participants’ retrospective comparisons of the two strategies were related to their choice of study strategy. Consequently, we used three semantic-differential measures: one comparing perceived mental effort, one for comparing perceived learning, and one for comparing perceived familiarity. Positive values on the created scales indicates that participants perceive an interleaved schedule as being more effortful, more effective for learning, and more familiar. A 3.5 indicates that participants did not show a preference between the two strategies.

Again, we first conducted individual logistic regressions to examine bivariate relations. For every point that participants perceived the interleaved schedule as being more mentally effortful (relative to blocking), their odds of choosing the method for future study declined 0.33 times (95% CI: [0.25, 0.43]), Wald *z* = −8.00, *p* < .001, Nagelkerke *R*^2^ = .39. By contrast, for every point that participants perceived interleaving as being more effective for learning, their odds of choosing it increased 15.94 times (95% CI: [7.88, 32.23]), Wald *z* = 7.71, *p* < .001, Nagelkerke *R*^2^ = .85, and for every point they perceived interleaving as being more familiar, their odds of choosing it increased 1.94 times (95% CI: [1.53, 2.46]), Wald *z* = 5.53, *p* < .001, Nagelkerke *R*^2^ = .16.

**Figure 5:**
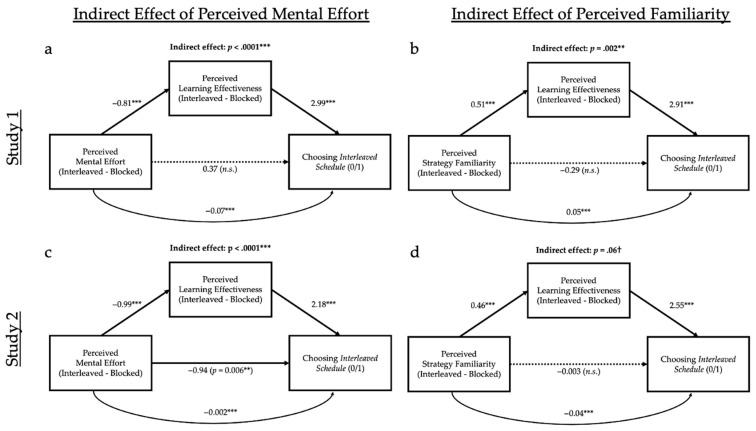
Bootstrapped mediation analyses for Study 1 for the unstandardized effects of mental effort (**a**) and familiarity (**b**) using the retrospective semantic-differential measures; bootstrapped mediation analyses for Study 2 for the unstandardized effects of mental effort (**c**) and familiarity (**d**) using the retrospective semantic-differential measures. † < .10, ** *p* < .01, *** *p* < .001.

Mediation analyses (see Figure 5a), replicating Kirk-Johnson et al. (2019) and supporting the misinterpreted-effort hypothesis, revealed that the more learners perceived interleaving as being effortful when compared to blocked learning, the less they perceived they learned (*t* = −17.31, *p* < .001, *R*^2^ = .48), and, consequently, the less likely they were to choose this method (*z* = 6.94, *p* < .001, partial *R*^2^ = .77; indirect effect: *z* = −8.00, *p* < .001). The direct effect of perceived mental effort on strategy choice was insignificant (*z* = 1.07, *p* = .28, partial *R*^2^ = .02), suggesting full mediation.

Critically, as hypothesized, strategy familiarity had a similar, indirect effect on choice (see Figure 5b), also mediated by perceived learning: the more that learners perceived the interleaving as being less familiar compared to blocked learning, the less they perceived they learned (*t* = −8.09, *p* < .001, *R*^2^ = .17), and, consequently, the less likely they were to choose this method (*z* = 7.08, *p* < .001, partial *R*^2^ = .83; indirect effect: *z* = −5.53, *p* < .001). The direct effect of perceived familiarity on strategy choice was insignificant (*z* = −0.89, *p* = .38, partial *R*^2^ = .01), suggesting full mediation.

#### 3.1.6. Free-Response about Changing Strategy Choice

Recall that, at the end of the experiment, participants were shown information about the normative effectiveness of interleaving and could describe whether this would change their choice. After viewing this information, participants either chose blocked (38.9%), interleaved (11.1%), or did not respond (50.0%) (see Table 1). We conducted an informal coding of the responses into four themes: participants who would not change, who would change, who might change, and who described an alternative method they could employ (e.g., a combination of the two strategies).

Among only the participants who originally chose blocked, the most common response was that participants said that they would not switch from blocked to interleaved even after viewing the normative data (63.7%). Participants who decided they would switch from the blocked to the interleaved strategy (20.8%) felt that they would now likely learn better with interleaving. Participants who were hesitant about switching from blocked to interleaved (9.6%) were unsure how to organize their work in a way that would be effective for interleaved study. Participants who said they would use an alternative study strategy (5.4%), such as a combination of both strategies, felt that they would likely start with blocked and then switch to interleaved for a more challenging study method.

Regarding only the participants who originally chose interleaved, again, the most common response was that participants said they would not change their study strategy (85.3%), which is perhaps unsurprising given that the normative data supported the use of an interleaving schedule. These participants often felt that they could more easily distinguish between the different bird families using an interleaved schedule. Participants who thought they would change their study strategy from interleaved to blocked (10.5%) often reported feeling that it would be less mentally effortful to distinguish between bird families with the blocked method. Participants who said they would use an alternative study strategy (1.2%), such as a combination of both strategies, felt that their study strategy approach would depend on the subject.

#### 3.1.7. Free-Response about Overall Experience

At the very end of the experiment, we also asked participants “Is there anything at all you’d like to share with us about your experience with the study today?” Of the 328 participants in Study 1, 47 (14.3%) provided a response (see Table 2). Participants’ responses were coded into six categories: hard to remember (difficulties with the study or had trouble remembering), productive (what participants learned from the study and their experience taking it), suggestion (points of confusion in the study and recommendations of what to change in future iterations), positive (overall positive response to the study), negative (generally negative response to the study), or other (did not fall into the previously defined categories).

For those who responded, participants who felt the information was hard to remember (4.3%) often said that the study was more difficult than they anticipated. Participants who had suggestions about the study (25.5%) mentioned that it would have been helpful to know what to look for when distinguishing between birds. Participants who had a positive experience with the study (40.4%) thought it was an interesting experiment. Participants who had a negative experience with the study (4.3%) stated that they did not enjoy the task or had difficulty paying attention. Other responses (4.3%) participants provided about the study typically included general comments about studying birds.

### 3.2. Study 2

#### 3.2.1. Strategy Choice

As with Study 1, when given the opportunity to choose either to use the blocked strategy or the interleaved strategy in the future, a majority of participants chose a blocked schedule (76.9%) versus the interleaved schedule.

#### 3.2.2. Immediate-Perception Questionnaire

Participants perceived the interleaved schedule (*M* = 3.54, *SD* = 1.20) as being, on average, 0.40 points more mentally effortful (95% CI: [0.51, 0.30] compared to the blocked schedule (*M* = 3.14, *SD* = 1.15), *t*(376) = 7.95, *p* < .001, *d* = 0.41. They also judged the interleaved schedule (*M* = 2.88, *SD* = 1.14) as being, on average, 0.49 points less effective (95% CI: [−0.36, −0.61]) for their learning when compared to the blocked schedule (*M* = 3.36, *SD* = 1.02), *t*(376) = −7.84, *p* < .001, *d* = −0.40. They also judged the interleaved schedule (*M* = 2.55, *SD* = 0.89) as being, on average, 0.50 points less familiar (95% CI: [−0.39, −0.63]) than the blocked schedule (*M* = 3.05, *SD* = 1.14), *t*(372) = −8.61, *p* < .001, *d* = −0.45.

#### 3.2.3. Retrospective Semantic-Differential Questionnaire

These comparisons showed a similar pattern to the immediate perceptions: participants judged an interleaved schedule as being significantly more effortful than a blocked schedule (*M* = 4.78, *SD* = 1.26, *t*(376) = 19.75, *p* < .001, 95% CI: [4.65, 4.91], *d* = 1.02), as significantly less effective for learning (*M* = 2.43, *SD* = 1.53, *t*(376) = −13.56, *p* < .001, 95% CI: [2.28, 2.59], *d* = −0.70), and as significantly less familiar (*M* = 2.58, *SD* = 1.65, *t*(375) = −10.83, *p* < .001, 95% CI: [2.41, 2.75], *d* = −0.56).

#### 3.2.4. Relation of Immediate Perceptions to Strategy Choice

We first examined the bivariate relationships between perceptions and strategy choices using individual logistic regression analyses. For every point that participants perceived the interleaved schedule as being more mentally effortful (relative to a blocked schedule), their odds of choosing it for future study declined 0.43 times (95% CI: [0.33, 0.58]), Wald *z* = −5.72, *p* < .001, Nagelkerke *R*^2^ = .15. By contrast, for every point that participants perceived the interleaved schedule as being more effective for learning, their odds of choosing it increased 4.79 times (95% CI: [3.32, 6.91]), Wald *z* = 8.39, *p* < .001, Nagelkerke *R*^2^ = .43. The relationship between familiarity and choice was marginal; for every point that learners perceived interleaving as being more familiar, their odds of choosing it increased 1.36 times (95% CI: [0.99, 1.86]), Wald *z* = 1.91, *p* = .06, Nagelkerke *R*^2^ = .05.

We next turn to our mediation analyses (see Figure 4c). Replicating both Kirk-Johnson et al. (2019) and Study 1, the more that learners perceived interleaving as being effortful, the less they perceived they learned (*t* = −11.56, *p* < .001, *R*^2^ = .26), and, consequently, the less likely they were to choose this method (*z* = 7.41, *p* < .001, partial *R*^2^ = .33; indirect effect: *z* = −5.72, *p* < .001). The direct effect of perceived mental effort on strategy choice was insignificant (*z* = −0.73, *p* = .47, partial *R*^2^ < .01), suggesting full mediation.

We also found marginal support for our familiarity-ease-of-learning hypothesis that strategy familiarity has a similar effect on choice (see Figure 4d), also mediated by perceived learning (*p* = .06): the more learners perceived the interleaving as being less familiar, the less they perceived they learned (*t* = 4.61, *p* < .001, *R*^2^ = .05), and, consequently, the less likely they were to choose this method (*z* = 8.32, *p* < .001, partial *R*^2^ = .42; indirect effect: *z* = 1.91, *p* = .06). The direct effect of perceived familiarity on strategy choice was insignificant (*z* = 0.30, *p* = .77, partial *R*^2^ = .04).

#### 3.2.5. Relation of Retrospective Semantic-Differential Perceptions to Choosing Final Strategy

Individual logistic regressions revealed that, for every point that participants perceived the interleaved schedule as being more mentally effortful (relative to blocking), their odds of choosing it for future study declined 0.13 times (95% CI: [0.09, 0.21]), Wald *z* = −9.28, *p* < .001, Nagelkerke *R*^2^ = .65. By contrast, for every point that participants perceived interleaving as being more effective for learning, their odds of choosing it increased 12.87 times (95% CI: [6.85, 24.16]), Wald *z* = 7.95, *p* < .001, Nagelkerke *R*^2^ = .84, and for every point they perceived interleaving as being more familiar, their odds of choosing it increased 1.73 times (95% CI: [1.48, 2.03]), Wald *z* = 6.77, *p* < .001, Nagelkerke *R*^2^ = .20.

Mediation analyses (see Figure 5c), replicating Kirk-Johnson et al. (2019) and our present Study 1, revealed the more learners perceived interleaving as being effortful when compared to blocked learning, the less they perceived they learned from this method (*t* = −27.80, *p* < .001, *R*^2^ = .67), and, consequently, the less likely they were to choose it (*z* = 6.48, *p* < .001, partial *R*^2^ = .59; indirect effect: *z* = −9.28, *p* < .001). The direct effect of perceived mental effort on strategy choice was also significant (*z* = −2.76, *p* = .01, partial *R*^2^ = .09), suggesting only partial mediation.

Further, as hypothesized, and replicating Study 1, strategy familiarity had a similar indirect effect on choice (see Figure 5d), also mediated by perceived learning: the more learners perceived interleaving as being less familiar when compared to blocked, the less they perceived they learned from it (*t* = −10.91, *p* < .001, *R*^2^ = .24), and, consequently, the less likely they were to choose it (*z* = 7.65, *p* < .001, partial *R*^2^ = .80; indirect effect: *z* = 6.77, *p* < .001). The direct effect of perceived familiarity on strategy choice was insignificant (*z* = −0.01, *p* = .99, partial *R*^2^ < .01), suggesting full mediation.

#### 3.2.6. Objective Learning

In Study 2, participants classified eleven novel images of each bird family as a post-test evaluation of their learning. A paired-samples *t*-test indicated that participants classified more exemplars correctly when bird families had been learned using the interleaved schedule (*M* = 60%, *SD* = 19%) versus those who learned using the blocked schedule (*M* = 55%, *SD* = 18%), *t*(376) = 3.44, *p* < .001, *d* = 0.18.

Thus, despite that the fact that most participants chose blocked practice for future learning and rated this strategy being, on average, more effective for learning, participants, on average, learned more with interleaved practice.

However, could some participants have chosen blocked practice because, despite the group-level means, blocked practice was more effective for them individually? We tested whether differences between the strategies in objective learning varied as a function of the strategy chosen for future use; however, an ANOVA revealed that the interaction between assigned strategy and preferred strategy was not significant (*p* = .28). Further, although the difference between strategies in objective learning was smaller for participants who chose blocking (*M* = 58% accurate for interleaved birds vs. *M* = 55% for blocked birds) than those who chose interleaving (*M* = 65% for interleaved birds vs. *M* = 58% for blocked birds), in no case did blocking outperform interleaving. Thus, there was no evidence that learners who prefer blocking were doing so because they were actually learning more from this method.

#### 3.2.7. Free-Response about Changing Strategy Choice

As with Study 1, at the end of the experiment, participants were shown information about the general effectiveness of interleaving and had the opportunity to explain whether this information would change their decision. After viewing this information, participants either chose blocked (38.5%), interleaved (11.5%), or did not respond (50.0%). We used identical criteria from Study 1 to code the responses.

Among the participants who chose blocking, the most common response, as with Study 1, was that participants said that they would not switch from blocked to interleaved even after viewing the normative data (60.3%). A total of 24.8% of participants decided that they would switch from the blocked strategy to the interleaved strategy, 2.8% of participants were unsure about changing from blocked to interleaved, and 2.0% of participants said they would use a different study strategy, such as a blend of both strategies. Participants had comparable reasons for their decision as to whether to change strategies (see Table 1).

Looking at the participants who originally chose interleaving, again and consistent with the findings of Study 1, the most common response was that participants said they would not switch their study strategy (80.4%). A total of 16.2% of participants felt they would change their study schedule from interleaved to blocked and had similar reasoning to participants of Study 1. None of the participants in Study 2 who originally chose interleaved said they would use an alternative strategy.

#### 3.2.8. Free-Response about Overall Experience

As in Study 1, at the end of the study, we asked participants to share their experience with the task. Of the 377 participants in Study 2, 53 (14.1%) provided a response. Participants responses were coded identically to Study 1.

Of those who responded, 24.5% were coded as hard to remember, 22.6% were coded as productive, 5.7% were coded as suggestion, 28.3% were coded as positive, 5.7% were coded as negative, and 13.2% were coded as other. Participants had similar responses to those of Study 1 (see Table 2).

## 4. Discussion

### 4.1. Review of Key Findings

In these two experiments, we examined participants’ perceptions of two contrasting study schedules, blocking versus interleaving, when learning to discriminate between bird images, as well as their decision about which strategy to use in the future. Consistent with prior research, we found that most participants preferred a blocked schedule over an interleaved schedule (Kirk-Johnson et al. 2019; Yan et al. 2016). Even when participants were explicitly told that ninety percent of learners learn better when items are interleaved (Kornell and Bjork 2008b; Kornell et al. 2010; Wahlheim et al. 2012; Yan et al. 2016; Zulkiply et al. 2012), most participants still believed that they were learning better with the blocked strategy, because it felt easier and more fluent. In Study 2′s post-test learning evaluation, contrary to participants’ perceptions, participants correctly classified more bird exemplars with the interleaved schedule versus the blocked schedule.

Why do most learners choose a blocked schedule? Replicating Kirk-Johnson et al. (2019), we found that participants perceived the interleaved schedule as being both more mentally effortful and less effective for learning the different bird families. Further, in both experiments, we found support for a mediation effect, such that the more mentally effortful participants perceived interleaving to be, the less they felt they were learning, and, consequently, the less likely they were to choose it for future study. We found a direct effect of perceived mental effort on strategy choice in only one case, Study 2′s retrospective semantic-differential measure, and, even then, there was also a significant indirect effect. Generally, we can deduce that people are not evading strategies because they find them to be effortful; they attempt to pick the optimal strategy, but, due to perceived mental effort, people mistakenly believe that a blocked schedule is best for learning.

New to this study, we found that habits have a similar impact on study strategy decisions, where the less participants perceive the interleaving schedule to be familiar, the less effective for learning they view it to be; thus, they choose a blocked schedule instead. In Study 1, we found a significant indirect effect of familiarity on study strategy choice, mediated by perceived learning, in both the immediate perceptions and the retrospective semantic-differential comparisons; in Study 2, the indirect effect was significant in the immediate perceptions, but only marginal (*p* = .06) in the retrospective comparisons. In sum, we found qualitative support for our hypothesis every time we looked for it and had statistically reliable evidence in all but one case^3^. By contrast, in no case did we find a direct effect of familiarity on choice. Together, these results support the familiar-ease-of-learning hypothesis over the familiarity-dependence hypothesis: learners did not choose familiar strategies simply because they were habitual, but because they believed they were learning more from them.

### 4.2. Misinterpreted-Effort Hypothesis and the Influence of Familiarity

As with Kirk-Johnson et al. (2019), we found support for the misinterpreted-effort hypothesis: that the effort associated with many strategies that are effective for long-term retention (i.e., the “desirable difficulties”) is perceived by learners as a sign of poor learning; consequently, they do not choose these strategies.

We now expand upon that work by showing how habits or familiarity can be integrated into that model: learners found the interleaved schedule to be less familiar, and this tendency predicted the degree to which they perceived interleaving as being more favorable for their long-term learning.

To our knowledge, this is the first known study to jointly examine the relation between habits and familiarity, perceived learning, and study strategy decisions. While previous work has established that people often favor information, habits, or study strategies that are likely to be familiar to them (Ariel et al. 2011; Ariel and Dunlosky 2013; Metcalfe et al. 1993), our study explicitly measured participants’ familiarity with the study strategies of interest.

Further, our study provides insight as to the mechanisms of why habits relate to differences in study behavior: familiar strategies are also perceived as being better for learning, such that the relation between strategy familiarity and strategy choice was mediated by perceived learning. Both our studies found full mediations; there was no effect of familiarity on strategy choice when controlling for learning perceptions. Similarly, in our open-ended debriefing, none of the participants indicated that they preferred the blocked schedule because it was more familiar to them. Learners do not choose strategies simply because they are familiar or easy, but rather because they interpret such strategies as being better for learning.

Together, the indirect effects of both mental effort and familiarity suggest that, in many cases—even low-stakes laboratory studies where there is no particular incentive to learn or master the material—learners make a sincere effort to choose the strategies that they think are most optimal for their learning. When their choices diverge from objective norms, it may be because influences such as familiarity or mental effort cloud their ability to determine which strategies are most effective. Indeed, these influences can be seen as highly similar, given that habits are not as mentally taxing compared to less habitual study practices (Ariel et al. 2009; Winne and Hadwin 1998). What may unite both effects is the strong influence of processing fluency on judgments. The effects of perceived effort relate to fluency: Song and Schwarz (2008) had participants read instructions in either easy-to-read or difficult-to-read fonts and found that participants judged easy-to-read fonts as being more fluent and were more willing to read them. Song and Schwarz (2009) also explored the relationship between familiarity and fluency and found that participants rated food additives as both more unfavorable and less familiar if they were difficult to pronounce when compared to easy-to-pronounce food additives. Ergo, both effort and familiarity have been linked to the fluency heuristic (Hertwig et al. 2008) where, if one exemplar is processed more fluently (i.e., is less effortful or is more familiar) than another, learners prefer it.

### 4.3. Objective Learning

Study 2 measured objective learning through a post-test where participants classified novel exemplars of each bird family. This post-test confirmed that, on average, interleaved learning is indeed more successful when learning to distinguish between bird families compared to blocked, consistent with previous meta-analyses (Brunmair and Richter 2019; Firth et al. 2021) showing that interleaving is better for learning than blocking when attempting to discriminate visual categories, and confirming this for our specific study, particular materials, and participant population. This test expands on the work of Kirk-Johnson et al. (2019), who did not measure whether interleaving yielded better learning of their materials. Despite this, most learners prefer blocked practice over interleaved. Thus, learners’ preference for blocking cannot be attributed to blocking being better for learning these bird families but is instead a genuine error in metacognitive monitoring.

While there was robust evidence for an interleaving effect (*p* < .001), the effect size had a relatively small magnitude in the present work (*d* = 0.18). It could be argued, then, that the failure of many participants to recognize the value of interleaving is of relatively little consequence, given interleaving would only yield modest gains in their learning. However, we argue that what is most striking is the diverging directions of participants’ perceived and actual learning: participants were not merely neutral between blocking and interleaving but viewed blocking as better for learning, even as interleaving yielded a reliably better post-test performance—a true “metacognitive illusion”. It is important to note that interleaving effects are most robust for short texts or for visual images, as used in the present study, and cannot be generalized to all tasks (Firth et al. 2021).

We also did not find any evidence that participants who chose a blocked schedule did so because those particular individuals did, in fact, learn better with blocking. The difference in participants’ learning under the two strategies did not significantly vary between participants who chose blocking for future study versus those who chose interleaving, and in neither case did blocking outperform interleaving. (See also Yan et al. 2016, for similar results.) This reinforces the conclusion that, for our study, the perception that blocking yields better learning is a metacognitive illusion that does not align with learners’ objective performance as seen in Study 2.

### 4.4. Perceptions: Causal or Correlation?

Are the relationships between perceived familiarity, perceived learning, and strategy choice causal in nature? Indeed, we can make a causal inference that interleaved practice results in more learning than blocked practice due to the use of random assignment—participants were randomly assigned to learn some bird families with interleaving and others with blocking, and the interleaved families were better learned in the Study 2 post-test. This conclusion also accords with prior meta-analyses of experimental studies of interleaving (Brunmair and Richter 2019).

However, for the variables in mediation analyses, this evidence is only correlational. The more that people rate interleaving as being effective for learning, the more likely they are to choose this, but we do not definitively know that one causes the other. Nevertheless, we can rule out some alternative explanations for the correlation. For example, the direction of the causation cannot run in reverse—it cannot be that the choice to interleave causes people to perceive it as better for learning because the immediate perception rating occurred before the choice of strategy. Further experimental work has suggested that the influence of JOLs (i.e., monitoring) or strategy choices (i.e., control) is, in fact, causal (e.g., Metcalfe and Finn 2008).

Another possibility is that participants’ perceptions and study strategy decisions were influenced by—and perhaps an artifact of—the act of having to rate study strategies for their perceived learning and other properties. However, Ariel and colleagues found that, in most cases, having students make JOLs throughout an experiment did not result in improved comprehension compared to reading the presented material (Ariel et al. 2021). (In contrast, learning gains were visible when participants were asked to recall information before making a JOL; in the present work, the post-test came only after all perception measures had been collected.) Similarly, to test if perception ratings influence learners’ perceptions in the present paradigm, Kirk-Johnson et al. (2019) contained an additional experiment that excluded all perception ratings. In their Study 4, participants solely experienced two strategies (restudying or retrieval practice) and chose which strategy they would use in the future. Kirk-Johnson et al. (2019) found that people continued to choose retrieval practice at essentially the same frequency, regardless of whether they filled out a perception questionnaire earlier in the experiment. Hence, we can reasonably conclude that, in general, participants’ perceptions and choices in this paradigm are not an artifact of the rating procedure.

### 4.5. Open-Ended Items

To provide supplemental, qualitative insight into participants’ subjective experience in the studies, we also included open-ended questions in the experiments after debriefing them as to the effectiveness of interleaved practice. Despite being informed that blocking is not as effective as interleaving, approximately two-thirds of participants still would not change their decision from blocking to interleaving in future study.

These participants often justified their choice in the debriefing by reporting that that they preferred blocking because it felt easier and less mentally straining compared to interleaved practice. This is broadly consistent with our claims regarding the relevance of perceived effort in influencing study decisions; on the other hand, our mediation analyses suggest that participants’ perceived learning better predicts their study strategy choice, rather than a direct effect of mental effort on learners’ study strategy decision. Participants may not have been able to fully describe why they prefer one strategy over another; this may be due to limitations with how deep people’s introspection is regarding self-report assessments.

Some participants suggested using a combination of both strategies. While this is an intuitively appealing approach, prior research has found that it is not necessarily any better than pure interleaving (Yan et al. 2017).

Other participants were also hesitant about switching from blocking to interleaving because they were uncertain about how to organize their work to effectively use an interleaved schedule in their studies. We know that students are often not taught optimal study methods, so it makes sense that they are uncertain how to implement the most effective study practices. Kornell and Bjork (2007) also note that, in many cases, students make decisions because of practical or logical constraints, such as a limited timeframe. This further emphasizes a need for efficient studying to be taught in formal education or other contexts, since at least some learners experience intuitive barriers about how to actually use normatively effective strategies (i.e., “How do I do it?”).

It would be ideal to instruct learners on good study habits and have them use those instead of suboptimal ones. However, given the influence of familiarity and habits on perceived learning (i.e., participants find blocked more familiar and habitual and feel they are learning more), and the results of the present study and related studies (Kirk-Johnson et al. 2019; Kornell and Bjork 2008b; Kornell et al. 2010; Wahlheim et al. 2012; Yan et al. 2016; Zulkiply et al. 2012), this likely would not be enough in practice. If a strategy feels hard or unfamiliar, learners probably will not think that it is effective even if they are told it is. This could be one reason why interventions to teach people specific strategies have only had limited success (Kornell and Bjork 2008b; Kornell et al. 2010; Wahlheim et al. 2012; Yan et al. 2016; Zulkiply et al. 2012).

### 4.6. Limitations and Future Directions

The present studies also contain some limitations. Our experiments only contained undergraduate psychology students, and thus cannot necessarily be generalized to the broader population. We do note that Kirk-Johnson et al. (2019) found that the support they obtained for the misperceived-effort hypothesis from this paradigm could be generalized from college students to the general population, but that study did not test the role of familiarity.

Additionally, we examined only one self-regulated learning decision: how to order to-be-learned material, i.e., interleaved versus blocked schedules. However, learners still need to make other study strategy decisions, such as when to study (e.g., massed versus distributed practice) (Baddeley and Longman 1978; Benjamin and Bird 2006; Pyc and Dunlosky 2010; Son 2004, 2010; Toppino et al. 2009), how long one should study (e.g., one time or many times) (Karpicke and Roediger 2007; Karpicke 2009; Kornell and Bjork 2008b; Postman 1965; Pyc and Rawson 2012; Rawson and Dunlosky 2011; Son and Metcalfe 2000; Tullis and Benjamin 2011; Vaughn and Rawson 2011), or how to study (e.g., retrieval practice versus restudying) (Carpenter et al. 2008; Karpicke and Roediger 2008; Karpicke 2012; Roediger and Karpicke 2006). Moreover, in a typical formal education setting and beyond, learners do not usually make a binary decision between two contrasting study strategies but instead have a multitude of choices about what, when, how, how long, and how often to study. Kirk-Johnson et al. (2019) did extend this paradigm to the choice of retrieval practice versus restudy and found evidence supporting the misinterpreted-effort hypothesis that was extremely similar to that obtained for the choice of blocking versus restudying—but, again, familiarity was not tested.

Our results reveal that learners used both the ease-of-processing heuristic to translate mental effort into perceived learning and the familiarity heuristic to translate perceived familiarity into perceived learning, but it is uncertain how and when these heuristics emerge. Future work could investigate whether our results can be replicated across multiple stages of development, from childhood to adulthood. By evaluating learners’ perceptions of mental effort, familiarity, and learning over time, we can better understand when these heuristics emerge. If we can pinpoint a general time range in which these patterns appear, we can carry out interventions to restructure this train of thinking (e.g., by reframing the misconceptions of mental effort indicating poor learning and/or that familiarity indicates that information is well-known).

The last twenty years have seen a sharp increase among learning and memory researchers in the educational ramifications of cognitive science. The findings from these experiments could be applied to education to highlight the role of desirable difficulties (Bjork 1994; Bjork and Bjork 1992; Schmidt and Bjork 1992) and promote more beneficial study habits within self-regulated learning. We note that our studies have solely focused on the perspective of the learner, but teachers may have similar misconceptions about more mental effort leading to less efficient learning. Indeed, one of the reasons that blocked practice feels more familiar and habitual to most learners is that blocking is typically used by teachers and professors in formal education contexts (Roediger and Pyc 2012). Thus, it would also be valuable to investigate educators’ perceptions of mental effort, learning, and familiarity with study strategies such as blocked and interleaved. By bringing educators’ attention to the positive impact of effortful learning and promoting an understanding that familiar study strategy habits are not necessarily the most beneficial, more effective teaching methods can be implemented into the classroom. Consequently, we can bridge the gap between current scientific research knowledge and learners’ choice of self-regulated learning strategies.

### 4.7. Conclusions

Learners must make self-regulated learning decisions, such as the order in which to study material, but not all choices are created equal. Some strategies are suboptimal and do not result in the most effective learning and retention of information. Why do learners not use the most worthwhile study strategies? In our two experiments, we found evidence for the misinterpreted-effort hypothesis, whereby learners believe that the mental effort needed for studying is indicative of poor learning, and hence think that these strategies are inefficient and do not choose them for subsequent use, replicating Kirk-Johnson et al. (2019). Expanding on the work of Kirk-Johnson et al. (2019), we investigated the impact of habits and familiarity on study strategy decisions and found a similar pattern and support for our familiarity–ease-of-learning hypothesis: learners perceive unfamiliar strategies as yielding poorer learning and do not choose them for future learning. These results indicate multiple variables, including both effort and familiarity, that can contribute to the metacognitive illusions that interfere with people identifying and choosing the best study strategies in their self-regulated learning decisions.

## Figures and Tables

**Table 1:** Free-response regarding change in strategy choice.

Coded Response	Frequency of Response (%)		Example
	Study 1	Study 2	
Originally chose blocked			
Not change strategy	63.7	60.6	“No this does not change my strategy that I would use, it is easier for me to group it together.”
Switch to interleaved	20.8	24.8	“Yes, actually. Now that I think about it, studying them not grouped together helped me notice patterns between the types of birds.”
Hesitant about switching to interleaved	9.6	8.2	“I think it does, I usually group together but if 90% of people really do learn better without it being grouped together, maybe I should give it a try.”
Alternative study strategy	5.4	2.0	“I would personally start out studying with examples grouped together, and then move to examples that are not grouped together.”
Originally chose interleaved			
Not change strategy	85.3	80.4	“Having an unexpected pattern makes your brain remember things much better.”
Switch to blocked	10.5	16.2	“Yes, I think it will take less of a mental toll to differentiate between topics.”
Hesitant about switching to blocked	0.0	0.0	_
Alternative study strategy	1.2	0.0	“I [would] use a different strategy depending on the subject. For math, I think examples that are not grouped together is better, but for topics like biology and chemistry, I enjoy grouping up related topics.”

**Table 2:** Free-response feedback about study.

Coded Response	Frequency of Response (%)		Example
	Study 1	Study 2	
Hard to remember	4.3	24.5	“At the beginning of the study, I felt much more confident … At the end though, I found myself second guessing all my answers and being really unsure about what kind of bird I was looking at.”
Productive	21.3	22.6	“I really liked participating and learned the two different study methods and how not grouping them together fit best for me and my study habits.”
Suggestion	25.5	5.7	“I understand that the study might’ve had to commit to a purely visual learning style, but I think the hardest part is not knowing what differences I’m supposed to be looking for.”
Positive	40.4	28.3	“I found the study really intriguing and would definitely do more research in the future on this study.”
Negative	4.3	5.7	“I could barely focus when looking at the photos.”
Other	4.3	13.2	“Birds are a cool animal to study.”

## Data Availability

Data supporting the reported results can be found at DOI:10.17605/OSF.IO/74XV2 (https://osf.io/74xv2/). Access is available as of 11 August 2022.

## Notes

1 When participants with perfect scores were removed, 63% of practice items were answered correctly. Our results revealed a full mediation for perceived mental effort on perceived learning on strategy choice (direct effect: *p* = .93, indirect effect: *p* < .001, total effect: *p* <.001) and marginal support for our mediation for perceived familiarity on perceived learning on strategy choice (direct effect: *p* = .38, indirect effect: *p* = .002, total effect: *p* = .002).

2 When participants with perfect scores were removed, 63% of practice items were answered correctly. Our results revealed a full mediation for perceived mental effort on perceived learning on strategy choice (direct effect: *p* = .32, indirect effect *p* < .001, total effect: *p* < .001 and marginal support for our mediation for perceived familiarity on perceived learning on strategy choice (direct effect: *p* = .71, indirect effect: *p* = .07, total effect: *p* = .006).

3 It is not precisely clear why the indirect effect did not quite reach significance in the retrospective comparisons in Study 2 but, given that the two studies were identical up to the point of the ratings, it is unlikely to be due to methodological differences and more likely attributable to sampling error.

## Appendix A

### Appendix A.1. Immediate-Perception Assessments of Perceived Mental Effort, Perceived Learning, and Perceived Familiarity

Perceptions of Perceived Mental Effort

(1) How tiring was the last exercise?
  1 = Not at all, 6 = A lot
(2) How mentally exhausting was the last exercise?
  1 = Not at all, 6 = A lot
(3) How difficult was the last exercise?
  1 = Not at all, 6 = A lot
(4) How boring was the last exercise?
  1 = Not at all, 6 = A lot

Perceptions of Perceived Learning

(1) How likely are you to be able to distinguish between the types of birds?
  1 = Not very likely, 6 = Extremely likely
(2) How good do you think your memory for the different types of birds will be?
  1 = Not very good, 6 = Extremely good
(3) How effective was this exercise in helping you to distinguish between the types of birds?
  1 = Not very effective, 6 = Extremely effective
(4) How well did you learn to distinguish the types of birds?
  1 = Not very well, 6 = Extremely well

Perceptions of Perceived Familiarity

(1) How much did the last exercise resemble the structure of your classes?
  1 = Not well, 6 = Extremely well
(2) How familiar are you with the study strategy you used?
  1 = Not familiar, 6 = Very familiar
(3) How new is this study strategy to you? *
  1 = Not new, 6 = Very new
(4) How well did the last exercise match your study habits?
  1 = Not very well, 6 = Very well

* reverse scored

### Appendix A.2. Retrospective Semantic-Differential Comparison Assessments between the Blocked Schedule and the Interleaved Schedule

Perceptions of Perceived Mental Effort

(1) Which exercise required more mental effort?
  1 = Grouped together, 6 = Not Grouped Together
(2) Which strategy would be harder for other people?
  1 = Grouped Together, 6 = Not Grouped Together
(3) Which strategy was more enjoyable? *
  1 = Grouped Together, 6 = Not Grouped Together
(4) Which strategy was harder for you?
  1 = Grouped Together, 6 = Not Grouped Together

Perceptions of Perceived Learning

(5) Which birds do you think you’ll remember better?
  1 = Birds Grouped Together, 6 = Birds Not Grouped Together
(6) Which do you think is a more effective learning strategy for you?
  1 = Grouped Together, 6 = Not Grouped Together
(7) Which do you think is a more effective learning strategy for the average person?
  1 = Grouped Together, 6 = Not Grouped Together
(8) Which strategy would you use to study in the future?
  1 = Grouped Together, 6 = Not Grouped Together

Perceptions of Perceived Familiarity

(9) Which exercise better resembled the structure of your classes?
  1 = Grouped Together, 6 = Not Grouped Together
(10) Which method of studying was more familiar?
  1 = Grouped Together, 6 = Not Grouped Together
(11) Which exercise was newer to you? *
  1 = Grouped Together, 6 = Not Grouped Together
(12) Which exercise better matched your study habits?
  1 = Grouped Together, 6 = Not Grouped Together

* reverse scored
